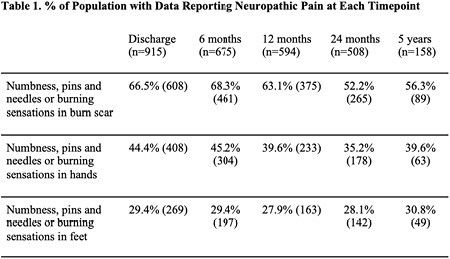# 89 Neuropathic Pain After Burn Injury: A Severe but Common Problem in Recovery

**DOI:** 10.1093/jbcr/irae036.088

**Published:** 2024-04-17

**Authors:** Eloise Stanton, Kara McMullen, Paul Won, Karen J Kowalske, Jeffrey C Schneider, Colleen M Ryan, Gretchen J Carrougher, Haig A Yenikomshian

**Affiliations:** Keck School of Medicine of USC, LOS ANGELES, CA; University of Washington, Seattle, WA; Keck School of Medicine, Los Angeles, CA; UT Southwestern Medical Center, Dallas, Dallas, TX; Spaulding Rehabilitation Hospital/Harvard Medical School, Boston, MA; Massachusetts General Hospital/Shriners Children's, Boston, MA; UW Medicine Regional Burn Center, Seattle, WA; University of Southern California, Los Angeles, CA; Keck School of Medicine of USC, LOS ANGELES, CA; University of Washington, Seattle, WA; Keck School of Medicine, Los Angeles, CA; UT Southwestern Medical Center, Dallas, Dallas, TX; Spaulding Rehabilitation Hospital/Harvard Medical School, Boston, MA; Massachusetts General Hospital/Shriners Children's, Boston, MA; UW Medicine Regional Burn Center, Seattle, WA; University of Southern California, Los Angeles, CA; Keck School of Medicine of USC, LOS ANGELES, CA; University of Washington, Seattle, WA; Keck School of Medicine, Los Angeles, CA; UT Southwestern Medical Center, Dallas, Dallas, TX; Spaulding Rehabilitation Hospital/Harvard Medical School, Boston, MA; Massachusetts General Hospital/Shriners Children's, Boston, MA; UW Medicine Regional Burn Center, Seattle, WA; University of Southern California, Los Angeles, CA; Keck School of Medicine of USC, LOS ANGELES, CA; University of Washington, Seattle, WA; Keck School of Medicine, Los Angeles, CA; UT Southwestern Medical Center, Dallas, Dallas, TX; Spaulding Rehabilitation Hospital/Harvard Medical School, Boston, MA; Massachusetts General Hospital/Shriners Children's, Boston, MA; UW Medicine Regional Burn Center, Seattle, WA; University of Southern California, Los Angeles, CA; Keck School of Medicine of USC, LOS ANGELES, CA; University of Washington, Seattle, WA; Keck School of Medicine, Los Angeles, CA; UT Southwestern Medical Center, Dallas, Dallas, TX; Spaulding Rehabilitation Hospital/Harvard Medical School, Boston, MA; Massachusetts General Hospital/Shriners Children's, Boston, MA; UW Medicine Regional Burn Center, Seattle, WA; University of Southern California, Los Angeles, CA; Keck School of Medicine of USC, LOS ANGELES, CA; University of Washington, Seattle, WA; Keck School of Medicine, Los Angeles, CA; UT Southwestern Medical Center, Dallas, Dallas, TX; Spaulding Rehabilitation Hospital/Harvard Medical School, Boston, MA; Massachusetts General Hospital/Shriners Children's, Boston, MA; UW Medicine Regional Burn Center, Seattle, WA; University of Southern California, Los Angeles, CA; Keck School of Medicine of USC, LOS ANGELES, CA; University of Washington, Seattle, WA; Keck School of Medicine, Los Angeles, CA; UT Southwestern Medical Center, Dallas, Dallas, TX; Spaulding Rehabilitation Hospital/Harvard Medical School, Boston, MA; Massachusetts General Hospital/Shriners Children's, Boston, MA; UW Medicine Regional Burn Center, Seattle, WA; University of Southern California, Los Angeles, CA; Keck School of Medicine of USC, LOS ANGELES, CA; University of Washington, Seattle, WA; Keck School of Medicine, Los Angeles, CA; UT Southwestern Medical Center, Dallas, Dallas, TX; Spaulding Rehabilitation Hospital/Harvard Medical School, Boston, MA; Massachusetts General Hospital/Shriners Children's, Boston, MA; UW Medicine Regional Burn Center, Seattle, WA; University of Southern California, Los Angeles, CA

## Abstract

**Introduction:**

Neuropathic pain (NP) is a poorly studied but common complaint of burn survivors and severely limits patients in regard to quality of life and function well after their burn injury. Currently, there is a paucity of data on the association between NP and its sequelae with burn survivors in the literature. As such, the purpose of this study is to better understand the incidence of NP and associated factors on a national scale using the Burn Model System (BMS) National Database.

**Methods:**

The BMS National Database was queried to identify burn patients responding to the primary outcome measure: numbness, pins and needles or burning sensations in three locations – (see Table 1) at enrollment, six months, 12 months, two years, and five years. The data were analyzed to determine the percent of the population reporting NP. Further subgroup analyses examined demographic and clinical characteristics associated with NP. Statistical analysis included non-parametric tests, Chi-square or Fisher’s exact, and regression analysis.

**Results:**

A total of 915 patients at discharge were included in analysis. At discharge, two-thirds of patients (66.5%, n=608) experienced NP, with over half still reporting NP at 5-year follow-up (Table 1). Patients with NP had significantly higher PROMIS-29 pain interference and itch scores at all time points after six months. NP patients had significantly higher anxiety, depression, and sleep disturbance PROMIS-29 scores and were significantly less able to participate in social roles at all follow-up points (Figure 1). Multiple logistic regression demonstrated male sex, %TBSA burn size, and moderate-to-severe pain on a zero to ten pain scale to be significant predictors of NP at 6-month follow-up (M sex: OR 1.9, p=0.042; %TBSA: OR 1.03, p=.009; moderate-to-severe pain: OR 5.4, p< 0.001). At 12-month follow-up, %TBSA burn size and moderate-to-severe pain were found to be significantly predictive of NP (%TBSA: OR: 1.03, p=.002; moderate-to-severe-pain: OR: 4.13, p=0.002). At 24-month follow-up, ethnicity, and employment status were significant predictors of NP (ethnicity: OR 3.1, p=0.033; employment status OR 3.0, p=0.016).

**Conclusions:**

The present study highlights the significant prevalence of NP in burn patients and the detrimental impacts on their physical, psychological, and social outcomes. The findings emphasize the importance of certain risk factors in NP and their role in enabling providers to provide more pointed and critical intervention. Moreover, the study supports a collaborative and comprehensive approach to treatment that accounts for the multifactorial nature of NP.

**Applicability of Research to Practice:**

The study emphasizes the importance of identifying specific risk factors to enable targeted interventions in clinical practice. A collaborative and comprehensive treatment approach that addresses both physical symptoms and psychosocial factors is crucial for improving long-term outcomes in burn patients with NP.